# Epidemiology of substance and opium use among adult residents of Tehran; a comprehensive report from Tehran cohort study (TeCS)

**DOI:** 10.1186/s12888-024-05561-1

**Published:** 2024-02-16

**Authors:** Farzad Masoudkabir, Akbar Shafiee, Amirhossein Heidari, Negin Sadat Hosseini Mohammadi, Kiarash Tavakoli, Arash Jalali, Sepehr Nayebirad, Farshid Alaeddini, Soheil Saadat, Ali Vasheghani-Farahani, Saeed Sadeghian, Vicente Artola Arita, Mohamamdali Boroumand, Abbasali Karimi

**Affiliations:** 1 Cardiac Primary Prevention Research Center, Cardiovascular Diseases Research Institute, Tehran University of Medical Sciences, Tehran, Iran; 2 Tehran Heart Center , Cardiovascular Diseases Research Institute, Tehran University of Medical Sciences , Tehran, Iran; 3 Faculty of Medicine, Tehran Medical Sciences, Islamic Azad University, Tehran, Iran; 4 Students’ Scientific Research Center (SSRC), Tehran University of Medical Science, Tehran, Iran; 5 Department of Emergency Medicine, University of California, Irvine, CA, USA; 6 Julius Center for Health Sciences and Primary Care, University Medical Center Utrecht, Utrecht, the Netherlands; 7 Department of Cardiovascular Research, Tehran Heart Center, North Kargar Ave, 1411713138 Tehran, Iran

**Keywords:** Substance use, Opium, Prevalence, Addiction, Iran, Tehran

## Abstract

**Background:**

The prevalence and burden of substance and opium use have increased worldwide over the past decades. In light of rapid population changes in Tehran, we aimed to evaluate the prevalence of opium and other substance use among adult residents in Tehran, Iran.

**Method:**

From March 2016 to March 2019, we utilized data from 8 296 participants in the Tehran Cohort Study recruitment phase (TeCS). We calculated the age-sex-weighted prevalence of substance use and the geographic distribution of substance use in Tehran. We also used logistic regression analysis to determine possible determinants of opium use.

**Result:**

We analyzed data from 8 259 eligible participants with complete substance use data and the average age of participants was 53.7 ± 12.75 years. The prevalence of substance use was 5.6% (95% confidence interval [CI]: 4.6- 7.1%). Substance use was more common in males than females (Prevalence: 10.5% [95% CI: 8.6- 12.6%] vs. 0.5% [95% CI: 0.2- 1.2%], respectively). The age-sex weighted prevalence of substance use was 5.4% (95% CI: 4.6-7.1%). Moreover, opium was the most frequently used substance by 95.8% of substance users. Additionally, we found that male gender (Odds ratio [OR]: 12.1, *P* < 0.001), alcohol intake (OR: 1.3, *P* = 0.016), and smoking (OR: 8.5, *P* < 0.001) were independently associated with opium use.

**Conclusions:**

We found that the prevalence of substance use in Tehran was 5.6%, and opium was the most frequently used substance. In addition, male gender, lower levels of education, alcohol, and tobacco consumption are the main risk factors for substance use in Tehran. Healthcare providers and policymakers can utilize our results to implement preventive strategies to minimize substance use in Tehran.

## Introduction

Substance use is a significant cause of disability and premature mortality globally [1–3]. It puts a huge burden on societies and governments significantly, including high economic costs, healthcare expenditures, lost productivity, increased violence, and poor treatment compliance [4]. The World Health Organization (WHO) estimates that in 2021, approximately 296 million people aged 15–64 engaged in substance use, reflecting a 26% increase over the preceding decade [5]. Cannabis constituted the predominant choice, with 219 million users, followed by opioids (60 million users) and methamphetamine (32 million users) worldwide [5]. However, the prevalence of substance use and the associated disease burden differed significantly across countries and varied among geographical regions [6]. According to the Global Burden of Disease report, the most substantial contributors to disability-adjusted life-years (DALYs) attributable to substance use were identified in the domains of substance use disorders (20.4 million DALYs), cancer (1.6 million DALYs), liver cirrhosis (4.8 million DALYs), and human immunodeficiency virus (HIV) (3.2 million DALYs) [6]. Moreover, substance use is responsible for almost 452,000 deaths annually.

Opioid dependence is one of the most common substance use disorders, with rates of 1.2% of the adult population worldwide, half of which is situated in South Asia or South-West Asia [5]. Moreover, opioids persist as the most lethal category of substances, responsible for approximately two-thirds of direct substance-related deaths, primarily through overdoses [5]. Consistently with global trends, substance use, particularly opium in Iran, has increased recently [7]. This is due to several factors, including the fact that Iran shares its border with Afghanistan, the world’s largest producer of opium, and Iran is a significant route for opium transport to Europe [8]. Another significant factor is Iran’s primary host of Afghan refugees (over three million). Given the historical prevalence of opium use among the Afghan population, prior evidence depicted the continuation of opium use among Afghan refugees in Iran [9]. These factors contribute to the escalated prevalence of opium use in Iran, facilitated by easy accessibility and low cost. In line with this, the WHO report illustrated Iran has the highest rate of opium users worldwide, and opium use disorder among Iranians is three times higher than global usage [10]. Moreover, previous literature demonstrated that around two million people in Iran are estimated to consume illicit substances, which equals 2.7% of the total population [11].

In accordance with robust evidence, substance use was strongly associated with mental, physical, and social health problems [12–14]. In spite of the high spectrum of problems associated with industrial substances, opium use as a traditional substance is related to several physical disabilities, including cancers and cardiovascular diseases [7].. Nevertheless, with the introduction of various health policies, substance use has still significantly increased in recent years [15–18]. In addressing this issue, health policymakers should be aware of the determinants influencing substance use to develop and implement proper prevention programs, potentially leading to a reduction in the incidence of substance use in society [19].

As of yet, a number of epidemiological and regional surveys have assessed substance use prevalence among Iranians, but their results were curtailed by small sample sizes and demographic differences among study populations [20, 21]. Furthermore, the prevalence of substance and opium use remains uncertain in Tehran, the capital of Iran, and available data are sparse and only encompass limited areas of the city. Additionally, previous investigations suggest that the male gender, younger age, socioeconomic status, as well as alcohol and tobacco use [22, 23]. Nevertheless, comprehensive data on predictors or determinants of substance and opium use in Tehran is still lacking.

Considering the ongoing population changes in Tehran, conducting a comprehensive study on substance use prevalence would be necessary to provide suitable insight into the current situation of substance use in Tehran for developing healthcare-related programs and governmental implementations [24, 25]. Therefore, as the first comprehensive investigation, we aimed to evaluate the prevalence and epidemiology of substance use, notably opium, in Tehran utilizing the recruitment phase data from the Tehran Cohort study (TeCS) data with a focus on identifying independent determinants associated with opium use.

## Methods

### Study design and participants

We examined data from TeCS, a population-based prospective study that involved adult residents of Tehran, the densely populated capital of Iran. Our study protocol has previously been published, providing a comprehensive description of the sampling methods and the recruitment phase of the TeCS study [26].. From March 2016 to March 2019, systematic random sampling was applied to recruit adults aged ≥ 35 years from all regions of Tehran. A total of 4 215 households, including 8 296 adults aged ≥ 35 years, enrolled in this study. Various demographic and medical data were collected, including past medical history, medications, familial history, anthropometric and physiological measurements, biochemistry, and laboratory tests. For this analysis, we included participants who had complete data on substance use. The TeCS protocol was reviewed and approved by the Deputy of Research and the Committee of Ethics at Tehran University of Medical Sciences (IR. TUMS.MEDICINE. REC.1399.074). All participants provided written informed consent upon enrollment in the study.

### Data collection and measurements

We interviewed every participant to collect data on demographic characteristics, such as age, marital status, ethnicity, educational level, employment status, medical history, pre-existing comorbidities, metabolic and behavioral risk factors, physical activity, level of education, alcohol and tobacco consumption, and substance use status. Several anthropometric indices were measured in all participants, including weight and height. Body mass index (BMI) was calculated by dividing weight by the height square (kg/m2). In addition, blood pressure (BP) was measured in a sitting position after at least five minutes of rest using a digital brachial cuff, and this assessment was conducted at least twice within a time frame of four hours based on recent guidelines [27].

### Definitions

We recorded the presence of diabetes, hypertension, dyslipidemia, chronic kidney disease, and cerebral vascular accident (CVA) in the participants based on history taking, medical documents, or medications. All information in the case of definitions of these conditions was completely explained in our previous reports from TeCS [28–30]. In brief, diabetes mellitus is characterized by a confirmed medical diagnosis of type 2 diabetes from healthcare professionals, fasting blood glucose (FBG) levels exceeding 126 mg/dl after an overnight fast lasting 8–12 h, or the utilization of glucose-lowering medications like oral hypoglycemic agents or insulin. Moreover, hypertension is defined as either a self-reported prior diagnosis of hypertension, systolic BP measurements equal to or exceeding 140 mmHg, diastolic BP measurements equal to or exceeding 90 mmHg, or the use of antihypertensive medications. Dyslipidemia is defined as suboptimal levels of high-density lipoprotein (HDL) cholesterol and/or elevated levels of triglycerides or low-density lipoprotein (LDL) cholesterol, accompanied by the use of lipid-lowering medications.

In the in-person interview questionnaire, we employed a subjective Likert scale to categorize average daily physical activity into low, intermediate, and high levels. We also asked them about their tobacco smoking status, whether current or past. We classified those who smoked cigarettes, pipes, or hookah occasionally or daily as current tobacco users. We defined former tobacco users as those who had quit smoking at least one month before the interview. We also asked about alcohol consumption in the last year, including any type of alcoholic drink.

We interviewed the participants using a comprehensive questionnaire that asked about their substance use patterns. We employed skilled interviewers who underwent comprehensive training in accordance with the Tehran Heart Center protocol to minimize data gaps and ensure clarity in our specialized inquiries, such as drug usage information. Our questionnaire included the type, frequency, and route of administration of substances. In the details, substances included opioids (such as heroin, morphine, opium, and opioid derivatives), cannabis, cocaine, and amphetamines. We considered an acceptable range of street names and custom labels associated with prevalent substances in Iran to make sure we identified all the substances in the participants. We defined current substance use as frequent use (on a daily basis) of any substance at least three months before the interview. We also asked them if they had quit using any substances based on their self-reports. Participants who did not report current substance consumption during the interview but provided a history of past substance use or indicated they had ceased using substances were classified as former substance users. Also, recreational users are individuals who have employed substances irregularly, notably opium, in traditional patterns, on specific occasions, or as a form of medication to alleviate pain and certain medical conditions. Participants who reported any level of opium consumption were categorized as opium users in the previous year.

### Statistical analysis

We reported categorical variables as frequencies and percentages for both men and women and compared them using the Chi-square test or Fisher exact test. We expressed continuous variables as means and standard deviations and compared them using the independent t-test for two groups or one-way ANOVA for more than two groups. We also calculated the total and sex-specific prevalence of substance use in our study population and the prevalence of opium and non-opium users. We weighted the prevalence of opium and substance use by age and sex based on the 2016 national census [31]. We evaluated the effect of baseline covariates on the odds of opium use utilizing a logistic regression model for ordinal dependent variables adjusting for age, sex, marital status, ethnicity, employment, level of education, BMI, diabetes mellitus, hypertension, dyslipidemia, CVA, chronic kidney disease, physical activity level based on Likert scale, tobacco use, and alcohol consumption. The adjusted association of the variables with opium use was reported through odds ratio (OR) with a 95% confidence interval (CI). We mapped the geographic distribution of substance use in Tehran based on the first three digits of the participants’ zip code using *shp2dta* and *spmap* modules in Stata software, release 14.2 (College Station, TX: Stata Corp LP.). We performed statistical analyses using IBM SPSS Statistics for Windows, version 23.0 (Armonk, NY: IBM Corp).

## Results

### General characteristics of the study population

**Fig. 1:**
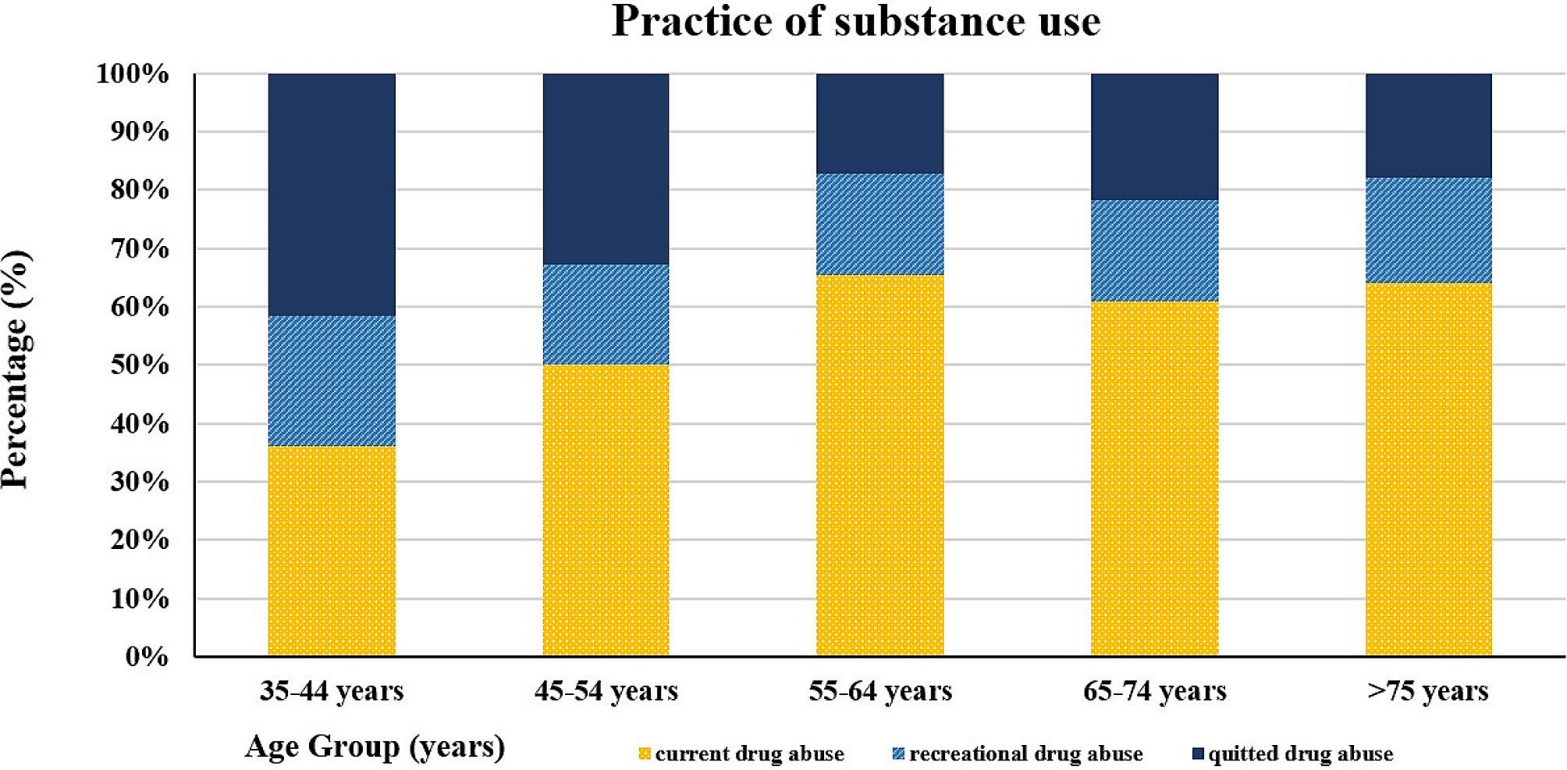
Practice of substance use in the different age groups

In this study, out of 8 296 adults aged ≥ 35 years, we assessed data from 8 259 eligible participants who had complete data on substance use (mean age: 53.7 ± 12.75, 54% women) and found a 5.6% prevalence of substance use among our study population in Tehran. The prevalence of substance use varied by age group, with the highest rate (7.6%) among participants aged 55 to 64. Males were much more likely to use substances than females, with a weighted prevalence of 10.9% (95% CI: 9.0–13.2) versus 0.6% (95% CI: 0.2–1.3). Opium use was the most prevalent substance in participants with a history of substance use, and the weighted prevalence was 5.3% (95% CI: 4.6–7.1), with a significant gender difference (weighted prevalence in males: 10.5% [95% CI: 8.6–12.6], weighted prevalence in females: 0.5% [95% CI: 0.2–1.2]). Moreover, among all subjects with a history of substance use, recreational and former patterns of use accounted for 1.0% and 1.5% of the study population, respectively (Figure 1). Table 1 summarizes all demographic, anthropometric, and medical characteristics comparisons between non-substance and substance users among our study population considering gender subgroups.

**Table 1:** Baseline characteristics of the eligible participants (divided by gender) in the Tehran Cohort Study according to substance use status

Demographic Characteristics^*^	Total (*n* = 8259)			Women (*n* = 4461)			Men (*n* = 3798)		
	Non-substance users (*n* = 7800)	Substance users (*n* = 459)	*P* value^#^	Non-substance users (*n* = 4435)	Substance users (*n* = 26)	*P* value	Non-substance users (*n* = 3365)	Substance users (*n* = 433)	*P* value
**Age, mean(SD), y**	53.6 (12.78)	56.4 (11.67)	< 0.001	52.9 (12.33)	60.5 (15.22)	0.017	54.6 (13.3)	56.2 (11.4)	0.008
**Age subgroups, n (%)**									
35–44	2233 (28.6)	84 (18.3)		1312 (29.6)	4 (15.4)		921 (27.4)	80 (18.5)	
45–54	2086 (26.7)	115 (25.1)		1119 (26.9)	6 (23.1)		895 (26.6)	109 (25.2)	
55–64	1811 (23.2)	148 (32.2)	< 0.001	1095 (24.7)	5 (19.2)	0.011	716 (21.3)	143 (33.0)	< 0.001
65–74	1131 (14.5)	84 (18.3)		593 (13.4)	5 (19.2)		538 (16.0)	79 (18.2)	
> 75	539 (6.9)	28 (6.1)		244 (5.5)	6 (23.1)		295 (8.8)	22 (5.1)	
**Ethnicity, n (%)**									
Fars	3798 (48.7)	226 (49.3)		2164 (48.8)	13 (50)		1634 (48.6)	213 (49.3)	
Azari	2318 (29.7)	130 (28.4)		1300 (29.3)	4 (15.4)		1018 (30.3)	126 (29.2)	
Gilak	314 (4.0)	18 (3.9)		192 (4.3)	1 (3.8)		122 (3.6)	17 (3.9)	
Lor	297 (3.8)	20 (4.4)		157 (3.5)	1 (3.8)		140 (4.2)	19 (4.4)	
Kurd	181 (2.3)	12 (2.6)	0.882	98 (2.2)	2 (7.7)	0.139	83 (2.5)	10 (2.3)	0.609
Mixed	640 (8.2)	42 (9.2)		397 (9.0)	3 (11.5)		243 (7.2)	39 (9.0)	
Other	198 (2.5)	9 (2.0)		96 (2.2)	2 (7.7)		102 (3.0)	7 (1.6)	
Immigrants/Refugees	48 (0.6)	1 (0.2)		30 (0.7)	0 (0.0)		18 (0.5)	1 (0.2)	
**Marital status, n (%)**									
Single	63 (0.8)	3 (0.7)		41 (0.9)	0 (0.0)		22 (0.7)	3 (0.7)	
Married	7547 (96.8)	445 (96.9)	0.937	4251 (95.9)	24 (92.3)	0.378	3296 (98)	421 (97.2)	0.436
Other	187 (2.4)	11 (2.4)		143 (3.2)	2 (7.7)		44 (1.3)	9 (2.1)	
**Education years, n (%)**									
Illiterate	542 (7.0)	43 (9.4)		419 (9.4)	8 (30.8)		123 (3.7)	35 (8.1)	
1–5 years	782 (10.0)	58 (12.6)	< 0.001	507 (11.4)	5 (19.2)	0.002	275 (8.2)	53 (12.2)	< 0.001
6–12 years	4008 (51.4)	284 (61.9)		2348 (53)	10 (38.5)		1660 (49.4)	274 (63.3)	
>12 years	2462 (31.6)	74 (16.1)		1160 (26.2)	3 (11.5)		1302 (38.8)	71 (16.4)	
**Employment**									
Unemployed	4669 (59.9)	179 (39.0)	< 0.001	3610 (81.4)	22 (84.6)	0.805	1059 (31.5)	157 (36.3)	0.046
Employed	3128 (40.1)	280 (61.0)		825 (18.6)	4 (15.4)		2303 (68.5)	276 (63.7)	
**Body mass index, mean(SD), kg/m2**	28.1 (4.83)	26.6 (4.51)	< 0.001	28.7 (5.17)	28.9 (4.16)	0.846	27.3 (4.21)	26.4 (4.5)	< 0.001
**Body mass index subgroups, n (%)**									
<20	195 (2.5)	30 (6.6)		98 (2.2)	0 (0.0)		97 (2.9)	30 (7.0)	
20-24.99	1930 (24.9)	142 (31.3)		1035 (23.6)	3 (13.0)		895 (26.8)	139 (32.3)	
25-29.99	3237 (41.8)	182 (40.1)	< 0.001	1649 (37.5)	12 (52.2)	0.629	1588 (47.5)	170 (39.4)	< 0.001
30-34.99	1712 (22.1)	81 (17.8)		1092 (24.9)	5 (21.7)		620 (18.5)	76 (17.6)	
> 35	663 (8.6)	19 (4.2)		520 (11.8)	3 (13.0)		143 (4.3)	16 (3.7)	
**Physical activity, n (%)**									
Low	1338 (17.3)	110 (24.4)		851 (19.3)	11 (42.3)		487 (14.6)	99 (23.3)	
Intermediate	4520 (58.3)	239 (53.0)	0.001	2677 (60.6)	10 (38.5)	0.01	1843 (55.3)	229 (53.9)	< 0.001
High	1890 (24.4)	102 (22.6)		888 (20.1)	5 (19.2)		1002 (30.1)	97 (22.8)	
**Alcohol use, n (%)**									
No	7157 (92.0)	341 (74.9)	< 0.001	4294 (97.0)	23 (88.5)	0.045	2863 (85.5)	318 (74.1)	< 0.001
Yes	622 (8.0)	114 (25.1)		135 (3.0)	3 (11.5)		487 (14.5)	111 (25.9)	
**Tobacco use, n (%)**									
Never	6244 (80.1)	94 (20.5)		4057 (91.5)	16 (61.5)		2187 (65.0)	78 (18.0)	
Quitted	273 (3.5)	60 (13.1)	< 0.001	46 (1.0)	0 (0.0)	< 0.001	227 (6.7)	60 (13.9)	< 0.001
Yes	1282 (16.4)	305 (66.4)		332 (7.5)	10 (38.5)		950 (28.2)	295 (68.1)	
**Diabetes mellitus, n (%)**									
No	6249 (81.5)	364 (81.4)	0.969	3582 (81.8)	17 (68.0)	0.113	2667 (81.1)	347 (82.2)	0.573
Yes	1418 (18.5)	83 (18.6)		796 (18.2)	8 (32.0)		622 (18.9)	75 (17.8)	
**Hypertension, n (%)**									
No	5614 (72.0)	325 (71.0)	0.636	3125 (70.5)	17 (65.4)	0.57	2489 (74.0)	308 (71.3)	0.235
Yes	2185 (28.0)	133 (29.0)		1309 (29.5)	9 (34.6)		876 (26.0)	124 (28.7)	
**Dyslipidemia, n (%)**									
No	5243 (67.2)	318 (69.4)	0.328	2893 (65.2)	18 (69.2)	0.67	2350 (69.8)	300 (69.4)	0.867
Yes	2556 (32.8)	140 (30.6)		1541 (34.8)	8 (30.8)		1015 (30.2)	132 (30.6)	
**Cerebral vascular accident, n (%)**									
No	7692 (98.6)	445 (96.9)	0.003	4380 (98.8)	25 (96.2)	0.276	3312 (98.5)	420 (97)	0.028
Yes	106 (1.4)	14 (3.1)		54 (1.2)	1 (3.8)		52 (1.5)	13 (3)	
**Chronic kidney disease, n (%)**									
No	7734 (99.2)	454 (98.9)	0.597	4401 (99.2)	26 (100.0)	> 0.0999	3333 (99.0)	428 (98.8)	0.605
Yes	66 (0.8)	5 (1.1)		34 (0.8)	0 (0.0)		32 (1.0)	5 (1.2)	
**Systolic blood pressure, mean (SD), mmHg**	121.6 (18.77)	124.3 (19.99)	0.005	119.2 (19.39)	120.1 (20.93)	0.813	124.8 (17.42)	124.6 (19.93)	0.852
**Diastolic blood pressure, mean (SD), mmHg**	80.8 (10.75)	79.6 (12.01)	0.033	80.8 (10.72)	81.1 (12.83)	0.892	80.9 (10.78)	79.5 (11.96)	0.026

Abbreviations: SD: Standard deviation

* Continuous variables with normal distribution are shown as mean ± standard deviation.

# *P* < 0.05 was considered statistically significant.

### Demographic assessment of participants with substance use

**Fig. 2:**
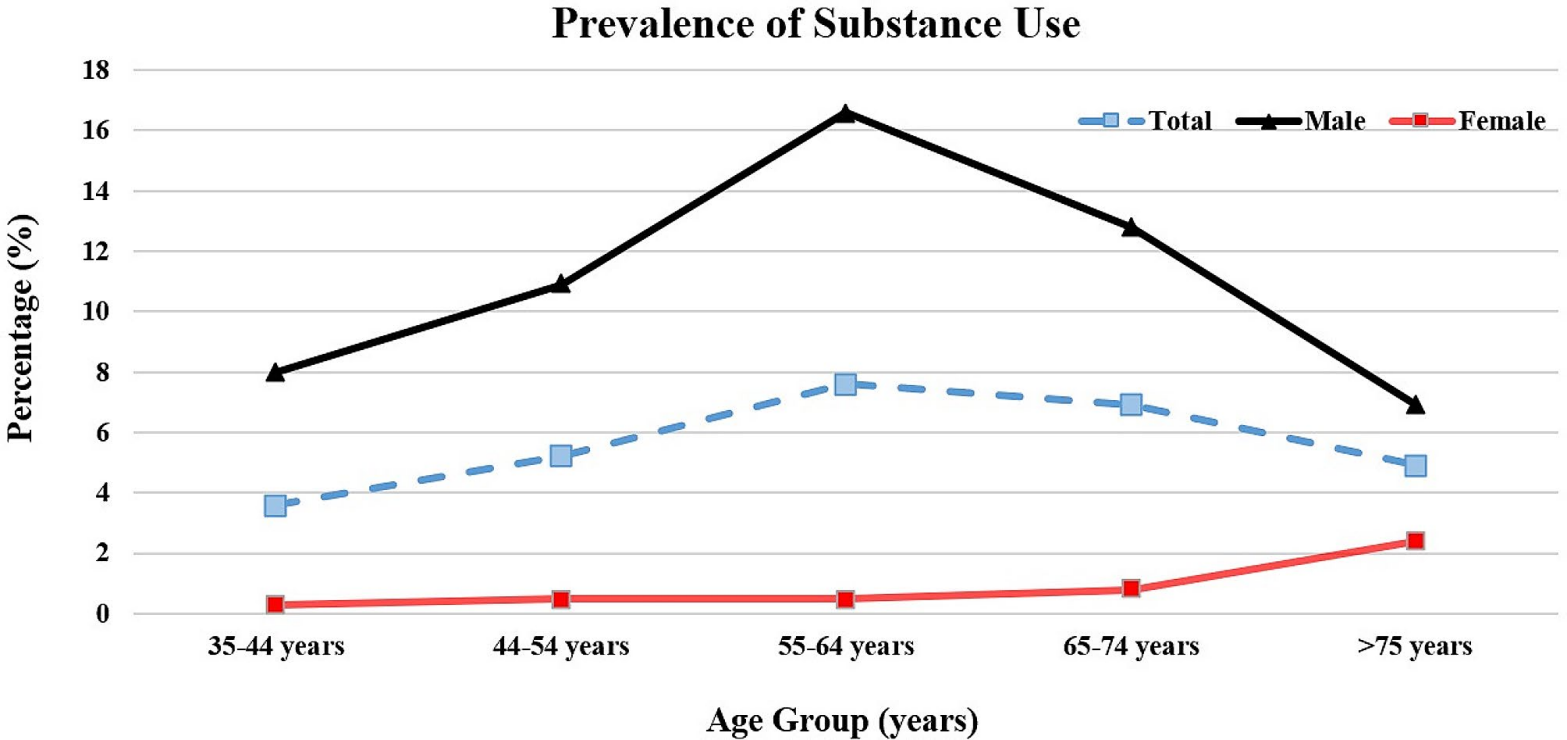
Comparison of substance use prevalence among both genders and different age groups

Based on our results, a total of 459 participants self-reported the use of substances during their lifetime. In detail, 95.8% (*n* = 440) of these participants consumed opium only, while 8.9% used other substances with or without opium (*n* = 42). Additionally, substance users were older than their peers in both genders (women: *P* = 0.017, men: *P* = 0.008). Regarding gender subgroups, substance use was also more prevalent among males in the 55–64 age subgroup (*P* < 0.001). Besides, older female subgroups were more likely to use substances (*P* = 0.011). Furthermore, substance use rates decreased with higher education levels (*P* < 0.001); however, most substance users had 6–12 years of education. In addition, employed participants had a higher rate of substance use (*P* < 0.001). Mainly, employed men were more likely to use substances than unemployed peers (*P* = 0.046). Besides, substance users had lower physical activity levels in both groups (*P* = 0.01, *P* < 0.001 for women and men, respectively), while non-substance users had higher BMIs, particularly among the men subgroup (*P* < 0.001). Participants who used substances also reported higher rates of alcohol (women: *P* = 0.045, men: *P* < 0.001) and tobacco use (*P* < 0.001 for both subgroups). Further, males with a history of substance were more likely to have experienced CVA (*P* = 0.028) among female participants; however, there were no significant differences. Compared to their peers, substance users had significantly higher systolic BP (*P* = 0.005); however, there were no statistical differences between gender subgroups. Additionally, Among men, substance users had significantly lower diastolic BP than non-users (*P* = 0.026). Marital status, diabetes, hypertension, dyslipidemia, and chronic kidney disease were similar in both groups. Figure 2 exhibited the frequency of substance use by gender and age group.

### Method and geographical pattern of use

**Fig. 3:**
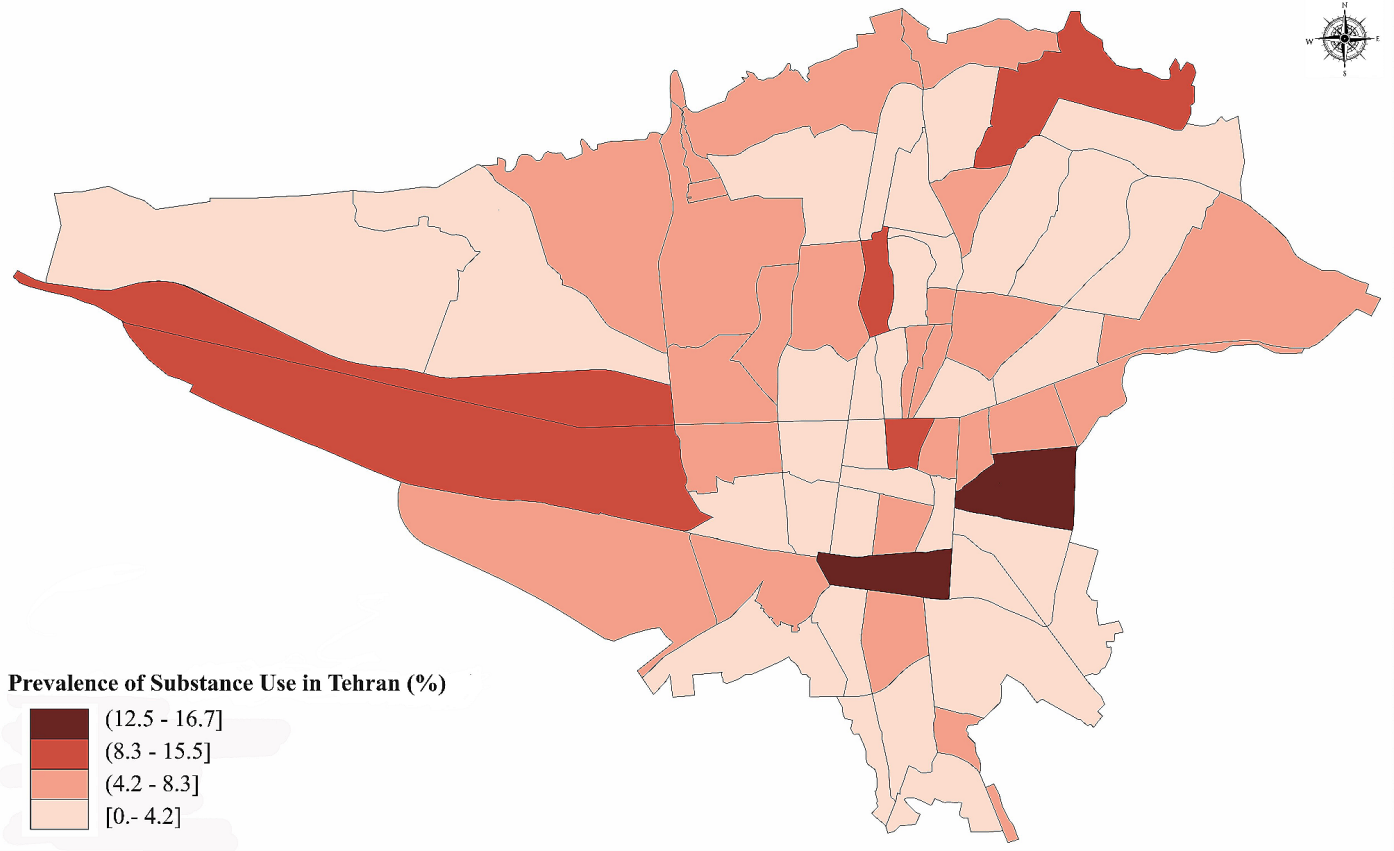
Geographic distribution of substance use in 22 districts of Tehran based on the first three digits of the residential zip codes. Suburban areas, particularly southern districts, are recognized for being prominent routes for drug trade and experiencing elevated levels of drug trafficking. More information about the details of the Tehran population and the distribution of participants from each district were indicated in our protocol [26]

Most participants (77.2%) used inhalation as their preferred mode of consumption, followed by ingestion (28.9%), and 7.33% reported using more than one mode. Substance use was higher in areas known for substance trafficking. Figure 3 displayed substance use prevalence in different regions of Tehran based on the first three digits of the zip codes.

### Determinants associated with opium use

With regards to Table 2, opium use was strongly associated with male gender (*P* < 0.001), alcohol use (*P* = 0.016), and tobacco use (*P* < 0.001). Among ethnicities, the Azeri population had the lowest opium use prevalence (OR: 0.75, *P* = 0.028). Higher education levels were protective against opium use. Compared to those without formal education, participants with 1–5 years of education had a 40% lower opium use risk (OR:0.60, *P* = 0.051), and those with more than 12 years of education had an OR of 0.15 (*P* < 0.001). Nonetheless, physical activity was inversely related to opium use (high physical activity: OR: 0.52, *P* < 0.001).

**Table 2:** Independent determinants of opium use in Tehran Cohort Study

Characteristics	Odds ratio	(95% CI)	*P* value*
**Age**	1.002	0.991–1.013	0.75
**Gender**			
Female	Reference		
Male	12.101	7.562–19.367	< 0.001
**Ethnicity**			
Fars	Reference		
Azari	0.750	0.580–0.970	0.028
Other	0.946	0.715–1.251	0.695
**Marital status**			
Single	Reference		
Married	1.178	0.263–5.287	0.831
Other	1.620	0.309–8.497	0.568
**Education years**			
Illiterate	Reference		
1–5 years	0.604	0.364–1.002	0.051
6–12 years	0.4555	0.294–0.707	< 0.001
>12 years	0.153	0.092–0.254	< 0.001
**Employment**			
Unemployed	Reference		
Employed	0.964	0.728–1.277	0.798
**Body mass index**	0.964	0.940–0.989	0.005
**Physical activity**			
Low	Reference		
Intermediate	0.642	0.484–0.851	0.002
High	0.526	0.376–0.736	< 0.001
**Alcohol use**			
No	Reference		
Yes	1.394	1.064–1.825	0.016
**Tobacco use**			
Never	Reference		
Quitted	6.131	4.184–8.984	< 0.001
Yes	8.536	6.493–11.220	< 0.001
**Diabetes mellitus**			
No	Reference		
Yes	0.869	0.640–1.178	0.366
**Hypertension**			
No	Reference		
Yes	1.232	0.920–1.649	0.161
**Dyslipidemia**			
No	Reference		
Yes	0.963	0.736–1.259	0.782
**Cerebral vascular accident**			
No	Reference		
Yes	1.632	0.800–3.331	0.178
**Chronic kidney disease**			
No	Reference		
Yes	0.742	0.256–2.150	0.583

Abbreviations: CI: Confidence interval

* *P* < 0.05 was considered statistically significant.

## Discussion

The current study aimed to determine the prevalence, patterns, and risk factors associated with substance use among adults 35 years old and greater in the Tehran population. As the first comprehensive investigation, our findings revealed a prevalence of 5.6% for substance use among the study population, with opioids being the most commonly used substance among our population. In addition, our results illustrated that opium use was independently associated with male gender, lower education levels, and alcohol and tobacco consumption.

Across the globe, substance use significantly contributes to major health-related problems, such as an escalation in infectious diseases such as hepatitis C and HIV, as well as an increase in physical and psychological disabilities [32–34]. Communities and governments are also confronted with significant challenges due to substance addiction, including the burden of substantial costs, high healthcare costs, functional impairment, violence, and criminal activities. According to the World Drug Report, a concerning rise in substance use has occurred over the past decade [35]. The report states that from 2006 to 2015, the percentage of substance users worldwide increased from 4.9 to 5.6%. This upward trend has considerably impacted the lives of millions worldwide, affecting approximately 275 million individuals.

There exists a long-term historical practice of employing opium as a traditional therapy in regions where opium is traditionally produced [36]. In Afghanistan, women engaged in the carpet weaving industry resort to opium for pain relief and to relax their children. Moreover, in Morocco, the opium poppy has been traditionally utilized to relieve pain, diarrhea, cough, and insomnia. Additionally, opium has conventionally been utilized as a pain-relieving substance, particularly among the elderly population in Iran [36]. A survey conducted in 2010 demonstrated that substance use in Iran is linked to tripled mortality rates among females and almost doubled mortality rates among males from 1990 to 2010 [33, 37].. In addition, based on a 2005 rapid assessment of substance use among the Iranian population, 1 200 000–1 800 000 people (2.4 − 2.65%) had a history of substance use [38]. Moreover, the National Survey on Substance Users and the National Survey on Mental Health estimated that substance use was 2.65% and 2.8%, respectively [39, 40]. Besides, the Iranian household mental health survey conducted in 2011 demonstrated that the prevalence of substance use is 2.09% and 2.44% based on DSM-IV and DSM-5, respectively [17]. Concerning related studies [11, 18, 41], our finding highlights the increasing trend of substance use in the Tehran population.

In accordance with global trends, males were more likely to use substances than females [18, 39, 42]. Moreover, the incidence of substance use exhibited a consistent upward trend in individuals aged 35 and above, reaching its apex within the 55–64 age group. This finding is alarming since the elderly population is more vulnerable to the adverse effects of substance use due to age-related physiological changes. Additionally, the association between lower educational level [18, 39], alcohol and tobacco consumption [42, 43], lower physical activity [43], and lesser BMI [43, 44] and substance use has been revealed in several studies. Prior investigations have also explained that the prevalence of substance use in Iran peaks at older ages compared to international reports [45, 46]. Albeit, there is a controversy over the results. Some studies support our findings by depicting a higher prevalence of substance use in the 5th and 6th decades of life [18, 39]; on the other hand, other studies considered the 3rd and 4th decades of life the highest prevalence of substance use [17]. Also, the prevalence of substance use has been reported in prior studies to be higher in previously married (divorced, separated, or widowed) individuals [18, 39]; However, our findings demonstrated no association between marital status and substance use [18].

In addition, it should be mentioned due to ongoing population changes and different ethnicities and populations within Iran, substance use, particularly opioid use, is prevalent at varying rates among regions. Regarding prior studies, the northern and southern parts of Iran have been found to have a higher prevalence of substance use [43, 47]. Evidence has also illustrated a higher prevalence of opioid use in Iran’s rural population than in its urban residents [48, 49]. As a novel finding of our study Tehran district substance users’ concertation in suburban areas, especially southern parts was significantly higher than in other areas. Additionally, we used separate analyses for each gender because the disparity in the prevalence of substance use between the sexes is evident in Middle Eastern populations. Since opium use and smoking are considered taboo in women, Middle Eastern women demonstrate considerably lower rates of substance use in comparison to their counterparts in Western regions [50]. Further, it is imperative to mention that the available data on substance use among women in Iran, particularly in Tehran, was limited, which also underscored the significance of our findings.

Conversely, to recent literature [51], our study also indicates that drug use is more prevalent among employed participants, mainly men. Concerning this observation, we can gain insight into the relationship between substance use and employment status. Various factors could contribute to this phenomenon, including stress and pressure associated with work demands [52]. In addition, substance use patterns may be influenced by the work environment itself. Further, the availability and social dynamics within the workplace can contribute to the greater prevalence of substance use among employed individuals, particularly men.

These results highlight that substance use, particularly opium, is a major public health challenge in the region that requires tailored interventions to mitigate its harms and disabilities. Moreover, regular surveillance of substance use trends and risk factors is essential for developing evidence-based policies and programs. Ultimately, a collaborative effort from policymakers, healthcare providers, and the community is needed to address the issue of substance use and its consequences in Iran and other affected countries.

### Strengths and limitations

The primary strength of this study lies in our provision of the first investigation and comprehensive data on substance and opium use in Tehran. The findings have the potential to serve as a foundational cornerstone for future studies and the development of public health plans and interventions in order to curb substance use, particularly opium, irregularly in Tehran. Similar to all cross-sectional studies, the current study also has some limitations, such as the presence of non-responders poses a challenge to accurately reporting the prevalence of substance and opium use in Tehran. Moreover, the observational design may be confounded by unknown variables. Furthermore, the limited number of females with substances in some analyses might impede the identification of significant differences. The broad objectives of TeCS necessitated the inclusion of only general inquiries about substance use status due to time constraints during data collection. Participants were interviewed for multiple questionnaires, and anthropometric measurements were conducted in a limited half-day session. Subsequently, detailed data on substance behaviors, such as the age of initiation and the exact amount of consumption, was lacking. Additionally, it is important to note that TeCS excluded adults aged ≥ 35 years,, potentially impacting our estimations, particularly for emerging substances like cannabis, which are believed to be more prevalent among the younger population.

## Conclusion

This study reported the prevalence, patterns, and associated determinants of substance and opium use in Tehran, Iran. We found that substance use prevalence was 5.6%, and opium was the most frequently consumed substance. Inhalation was the preferred mode of substance consumption, as well as substance use, was higher in areas known for substance trafficking. Independent determinants of opium use included male gender, alcohol and tobacco use, and lower education levels. In order to minimize Tehran’s substance use problem, healthcare sectors and policymakers should address our findings to develop preventive strategies and approaches, especially among vulnerable populations.

## Data Availability

The data underlying this article will be shared at a reasonable request to the corresponding author.

## Abbreviations

BMI: Body mass index
95% CI: 95% confidence intervals
CVA: Cerebrovascular accident
DALY: Disability-adjusted life-years
HIV: Human immunodeficiency virus
OR: Odds ratio
TeCS: Tehran cohort study
WHO: World Health Organization
